# Rationale and design of “Can Very Low Dose Rivaroxaban (VLDR) in addition to dual antiplatelet therapy improve thrombotic status in acute coronary syndrome (VaLiDate-R)” study: A randomised trial modulating endogenous fibrinolysis in patients with acute coronary syndrome

**DOI:** 10.1007/s11239-019-02014-5

**Published:** 2019-12-23

**Authors:** Ying X. Gue, Rahim Kanji, David M. Wellsted, Manivannan Srinivasan, Solange Wyatt, Diana A. Gorog

**Affiliations:** 1 Department of Postgraduate Medicine, University of Hertfordshire, Hatfield, UK; 2 Cardiology Department, East and North Hertfordshire NHS Trust, Hertfordshire, UK; 3 National Heart and Lung Institute, Imperial College, Dovehouse Street, London, SW3 6LY UK

**Keywords:** Acute coronary syndrome, Rivaroxaban, Endogenous fibrinolysis, Thrombosis, NOAC

## Abstract

Impaired endogenous fibrinolysis is novel biomarker that can identify patients with ACS at increased cardiovascular risk. The addition of Very Low Dose Rivaroxaban (VLDR) to dual antiplatelet therapy has been shown to reduce cardiovascular events but at a cost of increased bleeding and is therefore not suitable for all-comers. Targeted additional pharmacotherapy with VLDR to improve endogenous fibrinolysis may improve outcomes in high-risk patients, whilst avoiding unnecessary bleeding in low-risk individuals. The VaLiDate-R study (ClinicalTrials.gov Identifier: NCT03775746, EudraCT: 2018-003299-11) is an investigator-initiated, randomised, open-label, single centre trial comparing the effect of 3 antithrombotic regimens on endogenous fibrinolysis in 150 patients with ACS. Subjects whose screening blood test shows impaired fibrinolytic status (lysis time > 2000s), will be randomised to one of 3 treatment arms in a 1:1:1 ratio: clopidogrel 75 mg daily (Group 1); clopidogrel 75 mg daily plus rivaroxaban 2.5 mg twice daily (Group 2); ticagrelor 90 mg twice daily (Group 3), in addition to aspirin 75 mg daily. Rivaroxaban will be given for 30 days. Fibrinolytic status will be assessed during admission and at 2, 4 and 8 weeks. The primary outcome measure is the change in fibrinolysis time from admission to 4 weeks follow-up, using the Global Thrombosis Test. If VLDR can improve endogenous fibrinolysis in ACS, future large-scale studies would be required to assess whether targeted use of VLDR in patients with ACS and impaired fibrinolysis can translate into improved clinical outcomes, with reduction in major adverse cardiovascular events in this high-risk cohort.

## Highlights

- Impaired endogenous fibrinolysis is a novel, independent risk factor in patients ACS.
- Pharmacological modulation of endogenous fibrinolysis may improve outcome.
- The VaLiDate-R study will assess whether addition of low dose rivaroxaban to dual antiplatelet therapy can enhance endogenous fibrinolysis in ACS.

## Background

Antithrombotic treatment for acute coronary syndrome (ACS) beyond the acute admission, consists of dual antiplatelet therapy, comprising of aspirin together with a P2Y_12_ inhibitor [1, 2]. Following the results of the CURE study showing the benefits of adding the P2Y_12_ inhibitor clopidogrel to aspirin in patients with ACS [3], more potent P2Y_12_ inhibitors ticagrelor [4] and prasugrel [5], showed further reduction of ischaemic events compared to clopidogrel, albeit at the expense of higher bleeding risks. Beyond dual antiplatelet therapy (DAPT), trials have explored the concept of using triple (rather than dual) antithrombotic therapy to further reduce ischaemic events following ACS [6] with the addition to DAPT of cilostazol [7], vorapaxar [8], rivaroxaban [9] and dabigatran [10]. This has shown that whilst this approach can reduce recurrent ischaemic events, it significantly increases the risk of bleeding and therefore is not suitable for every patient [6].

Identification of patients at increased risk of future adverse thrombotic events would be highly desirable, since this group could be targeted with more potent antithrombotic medications, allowing use of less potent medications in low-risk groups to reduce bleeding and thereby increasing the net clinical benefit. Ideally, such therapy should be personalized to the individual, to achieve the greatest benefit at the lowest risk for the population. In order to offer personalized antithrombotic therapy, it is necessary to identify which patients remain prothrombotic, and at increased ischaemic risk, despite treatment with dual antiplatelet therapy (DAPT).

Following the onset of a thrombotic stimulus such as rupture or erosion of a thin-cap fibroatheroma, the likelihood of ACS and potential coronary occlusion is determined by the overall balance between factors which propagate thrombosis, mainly through enhanced platelet aggregation and activation of the coagulation cascade, and the effectiveness of the inherent defence mechanism of endogenous thrombolysis/fibrinolysis.

Whilst treatment with DAPT addresses the enhanced platelet reactivity in ACS, ongoing activation of the coagulation cascade and impaired endogenous fibrinolysis are unaffected by current DAPT. There is increasing evidence that impaired endogenous fibrinolysis is a strong predictor of residual cardiovascular risk in patients with ACS. Altered fibrin clot structure and increased resistance of the clot to lysis have been associate with myocardial infarction and stent thrombosis [11–14]. In the last 2 years, two large prospective studies have confirmed that impaired fibrinolysis in patients with ACS is a novel independent marker of increased cardiovascular risk [15, 16]. In a sub-study of > 4000 patients in the PLATO trial, assessment of fibrin clot lysis using a validated turbidimetric assay revealed that impaired fibrin clot lysis was an independent predictor of adverse outcome in ACS [15]. After adjusting for established cardiovascular risk factors, each 50% increase in lysis time was associated with cardiovascular death/spontaneous MI [Hazard ratio [HR] 1.17, 95% confidence interval (CI) 1.05–1.31; p < 0.01] and cardiovascular death alone [HR 1.36, 95% CI 1.17–1.59; p < 0.001]. Earlier work employing a point-of-care assay of whole blood fibrinolysis showed that some 23% of patients with non ST-segment elevation myocardial infarction (NSTEMI) exhibit impaired endogenous fibrinolysis (lysis time, LT ≥ 3000 s) despite DAPT, and this is predictive of recurrent adverse cardiovascular events [HR: 2.52, p < 0.04] and cardiovascular death [HR: 4.2, p = 0.033] over the subsequent year. with hazard increasing with increasing lysis time [17]. More recently, the RISK PPCI study from our group, involving nearly 500 patients with ST-segment elevation myocardial infarction (STEMI) showed that impaired endogenous fibrinolysis (LT ≥ 2500 s) detected in 14% patients on admission was strongly related to recurrent major cardiovascular events (HR 9.1, 95% CI 4.28–15.03, p = 0.001), driven by cardiovascular death and myocardial infarction [16].

### Pharmacological modulation of endogenous fibrinolysis

Unlike the enhanced platelet reactivity in these patients which reduced from admission to discharge, presumably reflecting the onset of effect of antiplatelet therapy, fibrinolysis in ACS patients appears unaffected by DAPT [15, 16]. Our group has previously investigated the effects of P2Y_12_ inhibitors on endogenous fibrinolysis and shown that these agents appear to have minimal impact on fibrinolysis [18]. In a small study, compared to baseline tests performed in the absence of anticoagulation, treatment with non-vitamin K antagonist oral anticoagulation appeared to favourably enhance endogenous fibrinolysis [19]. This finding is supported by the results of the ATLAS ACS-2 TIMI 51 study, showing that addition of very low-dose rivaroxaban 2.5 mg twice daily to DAPT in ACS patients significantly reduced the primary efficacy end point of the composite of death from cardiovascular causes, myocardial infarction, or stroke compared to placebo (9.1% vs. 10.7%, P = 0.02) but increased the risk of major bleeding and intracranial haemorrhage [9]. In individuals with stable cardiac or vascular disease, the COMPASS study demonstrated the benefit of Very Low Dose Rivaroxaban in addition to aspirin in reducing the composite of cardiovascular death, stroke, or myocardial infarction (4.1 vs. 5.4%, P < 0.001) albeit at a cost of increased bleeding [20].

### Study rationale

Measurement of endogenous fibrinolysis appears to identify patients who, despite DAPT, are at increased risk of recurrent adverse cardiovascular events. Additional pharmacotherapy to improve endogenous fibrinolysis may improve outcomes in high risk patients, whilst avoiding unnecessary additional pharmacotherapy and bleeding in low risk patients.

### Hypothesis

We hypothesize that in patients with ACS who demonstrate impaired fibrinolysis, use of Very Low Dose Rivaroxaban (VLDR), targeting the thrombin pathway, in addition to DAPT, will result in improved fibrinolytic profile, compared to patients taking DAPT alone.

## Methods

### Study principle and population

The VaLiDate-R study (ClinicalTrials.gov Identifier: NCT03775746, EudraCT: 2018-003299-11) is an investigator-initiated, randomised, open-label, single centre trial comparing the effects of ticagrelor, clopidogrel and clopidogrel combined with Very Low Dose Rivaroxaban on fibrinolytic status in patients with ACS. Patients admitted to hospital with ACS (including those with STEMI, NSTEMI and unstable angina) who fulfil the inclusion and exclusion criteria of the study will be eligible for inclusion.

The main objective of the study is to investigate the impact these pharmacotherapies on fibrinolytic status in patients with ACS.

### Inclusion and exclusion criteria

The VaLiDate-R study is an all-comers clinical trial enrolling patients presenting with ACS who meet the inclusion and exclusion criteria as set out in Table 1.

**Table 1:** VaLiDate-R inclusion/exclusion criteria

Inclusion	Exclusion
1. Male and female patients aged 18 years or over 2. Have a diagnosis of acute coronary syndrome requiring treatment with dual antiplatelet therapy 3. Be willing and able to understand the Participant Information Sheet and provide informed consent 4. Agree to comply with the drawing of blood samples for the assessments 5. Not meet any of the exclusion criteria	1. Male and female participants aged < 18 years of age 2. Patient unwilling or unable to give informed consent 3. Patients who might be pregnant or are breast-feeding 4. Active clinically significant bleeding 5. Patient who, in the opinion of the investigator, has condition considered to be a significant risk for major bleeding (such as current or recent gastrointestinal ulceration, presence of malignant neoplasm at high risk of bleeding, recent brain or spinal injury, recent brain, spinal or ophthalmic surgery, recent intracranial haemorrhage, known or suspected oesophageal varices, arteriovenous malformations, vascular aneurysms or major intraspinal or intracerebral vascular abnormalities) 6. Hepatic disease associated with coagulopathy and clinically relevant bleeding risk including cirrhotic patients with Child Pugh B and C 7. Patient with any contraindications to use of antiplatelet agents or anticoagulants 8. Hypersensitivity to the active substance or to any of the excipients listed in Summary of Product Characteristics (SmPC) of Rivaroxaban 9. Concomitant treatment with any other anticoagulants e.g. unfractionated heparin (UFH), low molecular weight heparins (enoxaparin, dalteparin, etc.), heparin derivatives (fondaparinux, etc.), oral anticoagulants (warfarin, dabigatran etexilate, apixaban etc.) except under specific circumstances of switching anticoagulant therapy or when UFH is given at doses necessary to maintain an open central venous or arterial catheter 10. Concomitant treatment of ACS with antiplatelet therapy in patients with a prior stroke or a transient ischaemic attack (TIA) 11. Patient with ongoing active alcohol or substance abuse or demonstrates signs or clinical features of active substance abuse 12. Patient with any major bleeding diathesis or blood dyscrasia at baseline (platelets < 70 × 10^9^/l, Hb < 80 g/l, INR > 1.4, APTT > x 2UNL, leucocyte count < 3.5 × 10^9^/l, neutrophil count < 1 × 10^9^/l) 13. Patient currently enrolled in an investigational drug trial

A two-stage consent process is adopted where an eligible patient will first consent to a screening blood test to identify impaired endogenous fibrinolysis before proceeding to a full consent to allow for randomisation and trial procedures. In cases of patients presenting acutely to the hospital with STEMI for PPCI, a delayed consent approach is undertaken, with ethical permission, whereby screening blood tests for assessment of thrombotic status are taken at the same time as standard of care blood samples on arrival, and patients are consented for the study after the PPCI procedure when they are stable and able to carefully consider the study. This is to ensure the consent process is fully informed (patients presenting with STEMI are unwell and often haemodynamically unstable, thus may not be in a suitable state to give full informed consent) and does not impact on the clinical care (explaining the study and obtaining full consent might cause delay to the emergency PPCI procedure) whilst not compromising the integrity of the sampling process (drugs such as heparin given during the procedure may affect the thrombotic status of patients). If after the emergency procedure the patient declines to participate or is unable to consent, the patient will not be included in the study. This will be recorded as a screen-fail. However, if the patient consents after the procedure, clinical information will be double checked to determine eligibility. If the thrombotic status is impaired and he/she fits the study criteria, written full informed consent will be obtained.

### Randomisation and study groups

Allocation of patients to one of the three study groups will be made by the use of a web-based block randomisation process. In subjects whose screening blood test shows that fibrinolytic status is impaired (LT > 2000 s), full written informed consent will be obtained, and patients will be randomised to one of 3 treatment arms in a 1:1:1 ratio: clopidogrel 75 mg daily (Group 1); clopidogrel 75 mg daily plus rivaroxaban 2.5 mg twice daily (Group 2); ticagrelor 90 mg twice daily (Group 3), in addition to standard therapy with aspirin 75 mg daily. For patients randomised to a new drug, for example patients initially treated with ticagrelor who are subsequently randomised to clopidogrel, a loading dose of the new medication will be given (clopidogrel 300 mg or ticagrelor 180 mg). The duration of rivaroxaban is 30 days after which the patient will stop rivaroxaban and will continue clopidogrel only or be switched to ticagrelor (including with loading), as decided by the clinical care team. Duration of P2Y_12_ inhibitor treatment will be determined by the clinical care team. All other treatments will be continued in accordance with standard of care, at the discretion of the clinical team.

### Study follow up

**Fig. 1:**
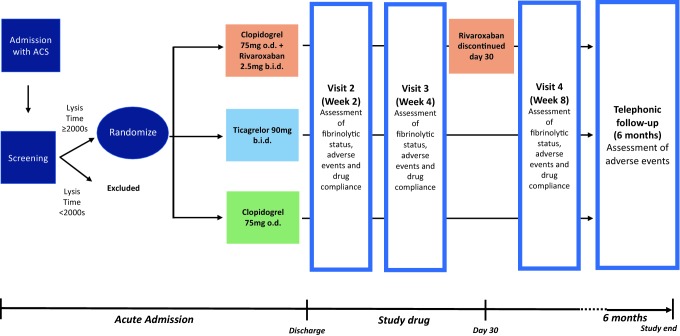
Outline of study and follow-up procedure

Patients will return for assessment and blood draw at 2 weeks, 4 weeks and 8 weeks to assess fibrinolytic status, record any adverse events and evaluate compliance. At each visit, subjects will be asked about compliance and asked to return any unused medication. Additionally, patients in the rivaroxaban arm will have Factor Xa levels assessed to confirm compliance during Visit 2 and 4, taken during the peak effect of rivaroxaban (between 2 and 5 h post-dose). A telephonic follow up will be performed at 6 months to assess for further clinical events. Figure 1 summarises the study flow and follow up procedures.

### Study related procedures

A single blood draw will be used for tests of thrombosis and thrombolysis. The first 10 ml blood will be used for the Global Thrombosis Test (GTT) assay and thromboelastography (TEG), and citrated plasma stored at − 80 ºC for subsequent analysis.

### Global thrombosis test

The Global Thrombosis Test (GTT) (Thromboquest Limited, London, UK) is point-of-care test of thrombotic status that utilises native (non-anticoagulated) blood to assess thrombosis (occlusion time) and thrombolysis (lysis time). A 4 ml blood sample is introduced into the cartridge and the measurement is fully automated. Blood flows under the influence of gravity at 37 °C, and is exposed to high shear stress, resulting in platelet activation. Further downstream, low shear and turbulent flow favours large platelet aggregate formation leading to the generation of thrombin and onset of thrombosis. Flow then carries these fibrin-coated platelet aggregates downstream leading to thrombotic occlusion. Downstream, a light-interruption sensor detects the blood drops: the instrument measures the time between two consecutive drops (*d*). At a predefined point (*d* ≥ 15 s) the occlusion time (OT, s) is detected. After a 15 s stabilisation period, during which the light sensor is inactive, the instrument detects restart of flow due to endogenous fibrinolysis and this is recorded as the lysis time (LT, s).

### Thromboelastography

Thromboelastography (TEG, Haemonetics Corporation, USA) is point-of-care technique measuring the viscoelastic properties of blood as it clots under low shear stress based on pin-and-cup technology. Whole blood is placed in the cup which oscillates 4° 45′ every 5 s while a pin on a torsion wire is suspended in the blood. As the viscoelastic strength of the clot increases, more rotation is transmitted to the torsion wire and is detected by an electromagnetic transducer. The kinetic changes during clot formation and lysis is shown graphically in the form of a thromboelastogram [21]. The measured parameters include the K index (a measure of platelet function), angle (a) which indicates the rate of fibrin-formation, maximal amplitude (MA) reflecting the platelet contribution to clot formation and finally clot lysis, which is assessed at 30 and 60 min, and recorded as Lysis 30 and Lysis 60.

### Rivaroxaban level

Rivaroxaban level in plasma samples will be measured using a specific chromogenic assay for anti-factor Xa activity with rivaroxaban calibrators by an investigator blinded to TEG and GTT results.

### Additional measurements

Additionally, citrated plasma will be stored at − 80 °C for future analysis of factorial components of the fibrinolytic cascade (including, but not limited to plasminogen activator inhibitor-1, tissue plasminogen activator and thrombin activatable fibrinolysis inhibitor) and turbidimetric assay of fibrinolysis which assess plasma clot lysis.

In patients randomised to rivaroxaban, anti-Factor Xa levels will be checked to assess compliance and measure plasma activity of rivaroxaban [22].

### Primary and secondary endpoints

The primary outcome measure is the change in fibrinolytic status as measured by Lysis Time (LT) using the GTT from admission to follow-up at 4 weeks post-discharge.

The secondary outcome measures are clinical events including re-intervention (further coronary angioplasty), major adverse cardiac events (composite of heart attack, stroke or death) and bleeding events evaluated by BARC criteria [23].

### Statistical considerations

We aim to compare the effects of three different antithrombotic treatment strategies on endogenous fibrinolysis. The study will seek to evaluate the extent to which Very Low Dose Rivaroxaban (Group 2) reduces LT compared to Group 1 and Group 3. Separate paired comparisons will be used to evaluate univariate effects between the study groups at 2 weeks, 4 weeks and 8 weeks. Similarly, the influence of Very Low Dose Rivaroxaban will be evaluated by considering change over time from baseline using paired comparisons. Multivariable models (mixed models) will be used to estimate adjusted difference for baseline patient characteristics, and to evaluate the influence of secondary variables on the treatment outcome. The effect of treatment group on major adverse cardiac events may be considered using Kaplan–Meyer, or Cox regression should sufficient events be determined to achieve reasonable power. Time to event is defined as the time from randomisation to the date of event and the censor date is defined as the last follow-up date for each participant i.e. 6 months from date of randomisation.

### Sample size calculation

Sample size was calculated using Stata version 15.1 (StatCorp, College Station, TX, USA). Assuming α = 0.05 for two sided tests, to detect a difference of LT = 90 s with a power 1 – β = 0.80, a sample size of 45 in each group would be required to detect a change in LT = 45 s. Accounting for a 10% drop-out, withdrawal and loss to follow up, we calculated 50 patients in each group, resulting in a sample size of 150.

### Ethical and regulatory aspects

The sponsor (East and North Hertfordshire NHS Trust, Stevenage, UK) has overall responsibility for the conduct of the study including assurance that the study is conducted in accordance to EU and international standards of Good Clinical Practice and International Conference on Harmonisation guidelines, applicable government regulations and Ethics policies and procedures. The monitoring of the study will be done by University of Hertfordshire. A trial steering committee has been established and is responsible for overseeing the good execution and administrative progress of the protocol. The Data Safety Monitoring Board is responsible for periodically reviewing and evaluating the accumulated study data for participant safety, study conduct and progress, and making recommendations to the steering committee concerning the continuation, modification, or termination of the trial.

## Status quo

The first patient in VaLiDate-R was enrolled in January 2019. As per April 2019, a total of 65 patients were screened and 25 patients were randomised. Recruitment is currently ongoing with the aim of randomising the last patient by the February 2020 and completing the study with 6-month follow up of the last patient by August 2020 and subsequent data lock.

## Conclusions and outlook

Achieving improved net clinical benefit with current pharmacological treatments of ACS have reached a plateau. Additional antithrombotic therapy in addition to antiplatelet therapy, in particular using Very Low Dose Rivaroxaban, has been shown to reduce adverse cardiovascular events but increase bleeding [9, 20].

Recent studies have identified impaired fibrinolysis in patients with ACS as a novel risk factor for recurrent adverse cardiovascular events, that is not affected by DAPT.

We propose to identify patients with ACS on DAPT who exhibit impaired fibrinolysis and assess whether the addition of Very Low Dose Rivaroxaban can favourably modify the fibrinolytic profile in these patients.

Through the VaLiDate-R study, we hope to (1) make use of a novel biomarker as a risk-stratification tool to identify patients who would benefit from more potent antithrombotic therapy and (2) assess the effect of additional low dose anticoagulation with rivaroxaban on endogenous fibrinolysis profile. If the addition of VLDR can bring about improvement in endogenous fibrinolysis in patients with ACS, future large scale studies would be required to assess whether the beneficial effects of enhanced fibrinolysis through use of VLDR in patients with ACS and impaired fibrinolysis can translate into improved clinical outcomes, with reduction in major adverse cardiovascular events.

## Abbreviations

ACS: Acute coronary syndrome
DAPT: Dual antiplatelet therapy
GTT: Global thrombosis test
LT: Lysis time
MACE: Major adverse cardiovascular event
NSTEMI: Non ST-segment elevation myocardial infarction
OT: Occlusion time
PPCI: Primary percutaneous coronary intervention
STEMI: ST-segment elevation myocardial infarction
TEG: Thromboelastography
VLDR: Very Low Dose Rivaroxaban
